# The novel sphingosine-1-phosphate receptors antagonist AD2900 affects lymphocyte activation and inhibits T-cell entry into the lymph nodes

**DOI:** 10.18632/oncotarget.18626

**Published:** 2017-06-27

**Authors:** Jing Song, Arie Dagan, Zhanna Yakhtin, Shimon Gatt, Sean Riley, Hugh Rosen, Reuven Or, Osnat Almogi-Hazan

**Affiliations:** ^1^ Department of Bone Marrow Transplantation, Hebrew University-Hadassah School of Medicine, Jerusalem, Israel; ^2^ Department of Biochemistry and Molecular Biology, Hebrew University-Hadassah School of Medicine, Jerusalem, Israel; ^3^ Department of Chemical Physiology, The Scripps Research Institute, California, USA

**Keywords:** sphingosine-1-phosphate, lymphocyte localization, S1PR, lymphocyte activation, T cells

## Abstract

Sphingolipid derivatives play key roles in immune cell migration and function. Synthetic sphingolipid analogues are used as therapeutics to intervene various inflammatory and malignant conditions. We hypothesize that different analogs have different effects on immune cells and therefore can be used as treatment for specific diseases. This study examines the properties of the novel synthetic sphingolipid analog, AD2900, and its effects on immune cell activation and lymphocyte localization in homeostasis. AD2900 is an antagonist for all sphingosine-1-phosphate (S1P) receptors. It demonstrates a significant inhibitory effect on the proliferation of activated human peripheral blood mononuclear cells, which is dependent on cAMP reduction and calcium signal transduction but not on phospholipase C activation. AD2900 causes a significant but reversible downregulation of S1P1 expression on the cell surface. AD2900 administration to C57BL/6J mice leads to the accumulation of T cells in the blood and spleen and in turn reduces T-cell number in the lymph nodes. Moreover, AD2900 treatment shows significant effects on the localization of T-cell subpopulations. These results demonstrate the key roles of S1P in T-cell trafficking in a steady state and suggest a potential clinical application for AD2900. Notably, this sphingolipid analog does not cause a severe lymphopenia. The clinical effect of AD2900 in hemato-oncologic diseases and immune-related diseases needs further investigation.

## INTRODUCTION

The lysophospholipid, sphingosine-1-phosphate (S1P), is accepted as a major regulator of immune cell trafficking and effector cell function [1, 2]. S1P naturally exists in a high concentration in the blood and lymph, but its concentrations are of magnitude lower in the tissues, including the thymus, lymph nodes, and interstitial fluids [3, 4]. Immune cells such as lymphocytes, hematopoietic progenitor cells, and dendritic cells employ the S1P gradient to regulate their trafficking [5]. S1P is also involved in tumor progression [6], neoplastic cell proliferation [7], migration [8], and resistance to chemotherapeutic drugs [9]. In some malignancies, S1P overexpression is associated with poor prognosis [10]. S1P can act either through the five isoforms of the S1P G-protein-coupled receptors (S1P1–S1P5) or function directly on intracellular targets, consequently prompting different downstream signaling cascades such as cell survival and Ca^2+^ mobilization [2, 11, 12]. The expression patterns of S1P receptors show diversity in the human immune system, but S1P1 is expressed by most immune cells. For example, T cells express S1P1 and S1P4 and B cells express S1P1 and S1P3, while natural killer (NK) cells express S1P1 and S1P5. The diverse but selective expression of S1P receptors by different immune cell populations provides lineage-specific regulation of effector functions.

T cells are central effector cells in the immune system. After exiting the thymus into the circulation, naïve T cells can be activated and differentiated into effector and memory T cells when encountering specific antigens presented by APC. After the activation process, which takes place primarily in the lymph node, the activated T cells return into the circulation and execute immune functions [13, 14]. Studies have shown that the S1P gradient facilitates lymphocytes to enter and egress between lymph nodes and the blood or lymph through the regulation of S1P1 expression on the surface of lymphocytes [15–17]. The egression of naïve T cells from the thymus also depends on the signaling of S1P1 [16, 18–22].

Given that sphingolipids and S1P show pleiotropic effects in immunity and malignancy, a number of synthetic innovative sphingolipid analogues were synthesized to be used as therapeutics for various conditions [1, 23]. The S1P receptor (1, 3, 4, and 5) agonist, FTY720, is the most studied S1P analogue [24–28]. FTY720 rapidly induces lymphopenia in the blood through the sequestration of lymphocytes in lymph nodes, but not in the spleen of humans or rodents. Lymphocyte retention in the lymph nodes, induced by FTY720, is associated with upregulation of CCR7 expression [25, 29] and the downregulation of S1P1 on the surface of lymphocytes [30]. Due to its anti-inflammatory properties, FTY720 was approved as a drug for patients with the relapsing form of multiple sclerosis [31, 32].

Based on the wide range of S1P effects on the immune system, we hypothesized that different sphingolipid analogues can be used to intervene diverse immune-related and malignant disorders. Our group synthesized a large number of non-natural sphingolipid analogues that are found to induce apoptosis in various cells [33–36]. Some of these analogues also efficiently inhibited cell proliferation induced by immune cell activation.

Herein, we describe the effects of the amine derivative, sphingolipid analogue, AD2900, on immune cell activation and lymphocyte localization in homeostasis. We found that AD2900 is an antagonist for all the S1P receptors 1 to 5 and demonstrates an inhibitory effect on immune activation of PBMCs by stimulating a part of the S1P signal transduction pathways. AD2900 also inhibits proliferation of several T-cell acute lymphoblastic leukemia (T-ALL) cell lines and a cutaneous lymphoma cell line. In addition, low-dose treatment with AD2900 leads to a significant but reversible downregulation of S1P1 cell surface expression on PBMCs. When administered to mice, AD2900 antagonistic activity leads to the inhibition of T-cell migration to lymph nodes, the reduction of circulating naïve T cells, and the accumulation of central memory-like T cells (Tcm) in the spleen as well as effector/effector memory-like T cells (Tef/em) in the blood. This influence on T-cell circulation demonstrates the key roles of S1P in T-cell trafficking in a steady state. The regulated localization of different T-cell subsets allows the cells to execute their specific functions. Importantly, AD2900 does not cause severe lymphopenia, in contrast to some of the other sphingolipid analogue treatments.

Taken together, the innovative sphingolipid analogue AD2900 was used to study the role of S1P in T-cell circulation during homeostasis and in lymphocyte activation. The unique properties of AD2900 may be used in the future to develop a drug for the immunotherapy of immune-related and oncologic/hemato-oncologic diseases.

## RESULTS

### Synthetic sphingolipid analogue AD2900 inhibits allogeneic activation of PBMCs

**Figure 1:**
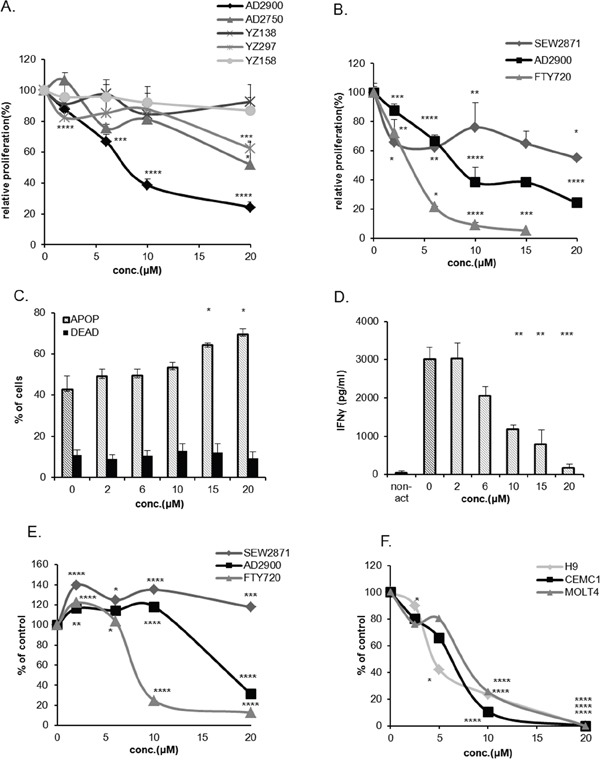
AD2900 inhibits human lymphocyte proliferation in allogeneic activation **(A, B)** The levels of proliferative response of human peripheral blood lymphocytes (PBMCs) after 1-day pretreatment with different concentrations of AD2900, AD2750, YZ138, YZ158, and YZ297 **(A)** or AD2900, FTY720, and SEW2871 **(B)** were tested by human allogeneic mixed lymphocyte reaction (MLR) assay, followed by a [3H] thymidine incorporation analysis. **(C)** Apoptosis of AD2900-treated activated PBMCs was tested by AnnexinV-propidium iodide (PI) FACS analysis following 1-day AD2900 pretreatment and MLR. All the samples were compared to the untreated PBMC control. **(D)** IFN-γ secretion from activated PBMCs was tested by ELISA following a 1-day AD2900 pretreatment in MLR. On the 5th day of hMLR, supernatants were collected and assayed for the level of IFN-γ. **(E)** Jurkat cell viability after a 1-day treatment with different concentrations of AD2900, FTY720, and SEW2871 was tested by XTT viability assay. **(F)** H9, CEM/C1, and Molt4 cell viability after a 1-day treatment with different concentrations of AD2900 was tested by XTT viability assay. Graphs summarize the results of at least three independent experiments. Results of Student’s *t*-test: *(P < 0.05, two-tailed test), ** (P < 0.01, two-tailed test), *** (P < 0.001, two-tailed test), **** (P < 0.0001, two-tailed test).

To select an analogue with potential anti-inflammatory activity, the effects of six of our unique synthetic sphingolipid analogues (all chemical structures can be found in Supplementary Figure 1A) on human immune cell activation were examined. Human PBMCs were cultured with the compounds for 1 day at different concentrations. The cells were then activated by PBMCs from a different donor in a mixed lymphocyte reaction (MLR) experiment. Among the tested analogues, AD2900 had the strongest dose-dependent inhibitory effect on PBMC proliferation with a mean of up to 76% ± 4% inhibition at the highest concentration (Figure 1A). The amine derivative, AD2900, is substituted with amino diols. Structurally, AD2900 has a hydroxymethyl group on carbon 2 and no phenyl substitution. These characteristics cause AD2900 to be more hydrophilic than other analogues. In addition, previous studies have shown that AD2900 has a higher lethal dose 50% (LD50), which is above 100 mg/kg, and it is stable at room temperature for at least 2 years (unpublished data). Based on these characteristics, AD2900 was selected for further investigation. Nuclear magnetic resonance (NMR) spectroscopy characteristic of AD2900 is shown in Supplementary Figure 1B. Next, we compared the effect of AD2900 on lymphocyte activation to the effect of commercial analogues FTY720 and SEW2817. Treatment with AD2900 for 1 day prior to an MLR test led to a compromised effect in comparison to FTY720 but a higher inhibitory effect than that with SEW2817 at concentrations of 6–20 μM as demonstrated in Figure 1B. Lower concentrations led to similar results when the analogues were sustained in the culture at the time of MLR (data not shown). The influence of AD2900 at different concentrations on the proliferation of non-activated PBMCs was also tested, which revealed no significant differences (Supplementary Figure 2A).

To better characterize the effect of AD2900 on activated PBMCs, the influence of AD2900 on apoptosis and cytokine secretion was tested. After 5 days of MLR, the cells were collected for apoptosis evaluation, while the supernatant was collected for ELISA test. Apoptosis was induced on the activated PBMCs only at higher concentrations of AD2900, above 10 μM (Figure 1C). Therefore, only a small part of the effect of AD2900 on the suppression of lymphocytic proliferation might be attributed to apoptosis. AD2900-induced inhibition of proliferation was accompanied by a reduced level of IFN-γ concentrations in the supernatant (Figure 1D). However, TNF-α concentration in the culture media was not influenced (Supplementary Figure 2B). Taken together, these results demonstrate that AD2900 affects lymphocyte activation, but this effect is mostly apoptosis independent.

There is abundant evidence that sphingolipid signaling plays a role in the progression of hematopoietic malignancies [8]. To examine the effect of AD2900 on the viability of T-ALL cells, we used the Jurkat cell line. In the XTT viability test, a 1-day treatment with AD2900 led to a reduced viability of the cells at concentrations higher than 10 μM (Figure 1E). Similar to the PBMC proliferation assays, the effect was lower than that with FTY720 treatment and higher than that with SEW2817. In other cancerous T-cell lines, CEM/C1, Molt4 (T-ALL), and H9 (cutaneous lymphoma), reduced cell viability could be detected even at lower concentrations of AD2900 (Figure 1F).

### Effect of AD2900 on PBMC proliferation is dependent on cAMP reduction and calcium signal transduction

**Figure 2:**
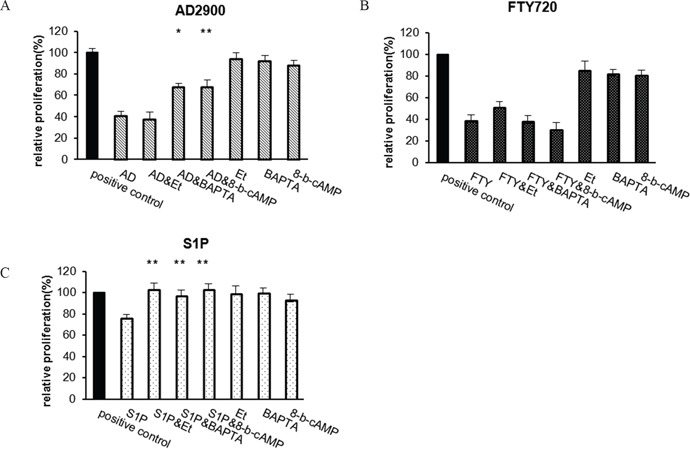
Effect of AD2900 on proliferation is dependent on cAMP reduction and calcium signal transduction The effect of Edelfosine (Et-18-OCH_3_) (Et), BAPTA-AM (BAPTA), or 8-bromo-cAMP (8-b-CAMP) on the inhibition of PBMC proliferation was tested in a 5-day MLR experiment. The cells were incubated with 1 μM Edelfosine, 0.2 μM BAPTA-AM, or 5 μM 8-bromo-cAMP for 1 hour before the addition of sphingolipid analogues, 2 μM AD2900 (AD) **(A)**, FTY720 (FTY) **(B)**, or S1P **(C)**. Significances are compared to AD2900/FTY/S1P without preincubation, respectively. Graphs summarize the results of at least four independent experiments. Results of Student’s *t*-test: *(P < 0.05, two-tailed test), ** (P < 0.01, two-tailed test).

The sphingolipid S1P also inhibits T-cell proliferation. It was previously demonstrated that S1P suppression of T-cell activation requires Ca^2+^ signaling, PLC activation, and cAMP reduction [11, 12]. To determine whether the effect of inhibition of AD2900 on PBMC proliferation is dependent on the same signal transduction pathways, we next examined the capacities of the intracellular Ca^2+^ chelator-BAPTA-AM, phosphatidylinositol-specific PLC inhibitor-Et-18-OCH3, and cell-permeate exogenous cAMP-8-bromo-cAMP to alter the suppressive effect of AD2900 and FTY720 on PBMC proliferation. S1P served as a positive control (Figures 2A, 2B, and 2C, respectively). PBMCs were pre-incubated for 1 hour with the inhibitors before the addition of 2 μM AD2900, FTY720, or S1P in the MLR experiment. The inhibitors and the analogues were present in the culture throughout the allogeneic activation. AD2900 suppression of PBMC proliferation was reduced from 59% ± 3% to 32% ± 7% by the exogenous cAMP. BAPTA-AM also partially reversed AD2900 suppression of PBMC proliferation from 59% ± 3% to 32% ± 3%, yet Et-18-OCH3 had no effect (Figure 2A). Interestingly, FTY720 suppression of PBMC proliferation was not nullified by any of these interventions (Figure 2B). As expected, S1P suppression was reversed by all three inhibitors (Figure 2C). These results confirm that AD2900 suppression of PBMC proliferation is dependent on cAMP reduction and calcium signal transduction rather than on the PLC signaling pathway, suggesting that the specific effect of AD2900 on PBMC activation is signal transduction dependent.

### AD2900 is an antagonist for sphingosine1- 1-phosphate receptors 1, 2, 3, 4, and 5

Since AD2900 is a sphingolipid analogue with properties that resemble those of S1P, we intended to examine its ability to interact with the S1P receptors. To delineate whether AD2900 can function as an agonistic or antagonistic compound to any of the S1P receptors, we used a high-throughput screening cell-based assay, employing cell lines stably transfected with human S1P receptors 1, 2, 3, 4, or 5 [37]. In this experiment, a compound with an IC50 or EC50 value ≤10 μM for a certain S1P receptor is considered active as an antagonist or an agonist, respectively. The results illustrate that AD2900 acts as an antagonist to all S1P receptors (Table 1, Supplementary Figure 3). Among these S1P receptors, AD2900 exhibits the highest antagonism property to S1P5 at a submicromolar level. No agonist activation was evident. Overall, these results show that AD2900 acts as a broad S1P receptor antagonist.

**Table 1:** AD2900 shows antagonistic activities against S1P1, 2, 3, 4, and 5

Compound ID	S1P1 Agonist EC50(μM)	S1P1 Antagonist IC50(μM)	S1P2 Agonist EC50(μM)	S1P2 Antagonist IC50(μM)	S1P3 Agonist EC50(μM)	S1P3 Antagonist IC50(μM)	S1P4 Agonist EC50(μM)	S1P4 Antagonist IC50(μM)	S1P5 Agonist EC50(μM)	S1P5 Antagonist IC50(μM)
AD2900	>50	3.8	>50	3.2	>50	6.2	>50	5.9	>50	0.405

Antagonistic and agonistic properties of AD2900 to S1P1–5 were tested using S1P1, S1P2 CRE-BLA CHO cell lines, S1P3 NFAT-BLA CHO cell line, or S1P1, S1P4, S1P5 TANGO U-2 OS cell lines in a high-throughput screening experiment. IC50 values (μM) or EC50 values (μM) of AD2900 were measured. Data represent mean 6 SEM obtained from three independent experiments. Abbreviations: CHO, Chinese Hamster Ovary; NFAT, nuclear factor of activated T-cell; BLA, beta lactamase; CRE, the cAMP Response Element promoter; U-2 OS, human osteosarcoma.

### AD2900 regulates the expression of sphingosine-1-phosphate receptor1 (S1P1) and CCR7 on PBMCs

**Figure 3:**
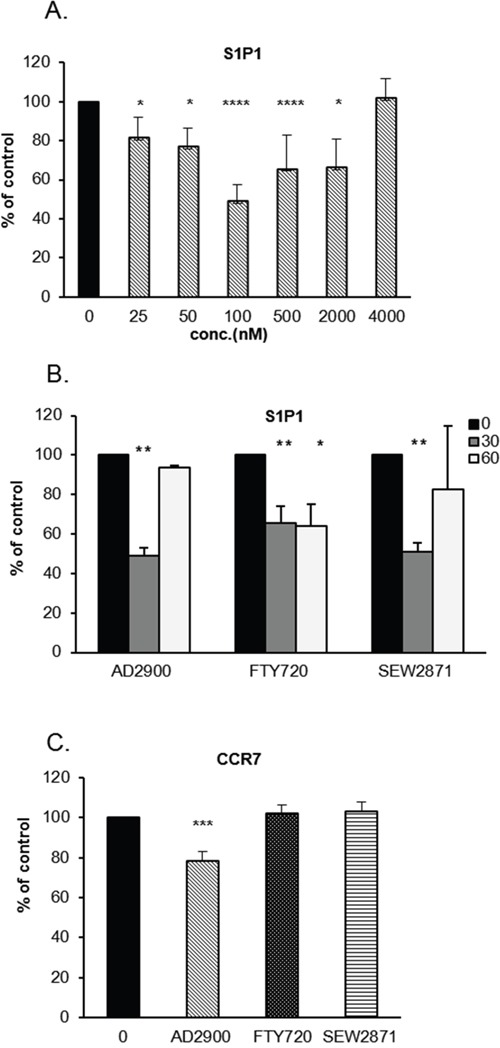
AD2900 downregulates the percentage of S1P1- and CCR7-expressing cells in PBMCs **(A, B)** The percentages of S1P1-positive PBMCs after the treatment with different concentrations of AD2900, FTY720, or SEW2871 and at different time points were examined by FACS analysis. S1P1 expression was tested in PBMCs after a 30-min treatment with AD2900 at different concentrations **(A)** or after a 30-min or 60-min treatment with 100 nM AD2900, FTY720, or SEW2871 **(B)**. **(C)** The percentage of CCR7-positive PBMCs was tested by FACS analysis after a 30-min treatment with 100 nM AD2900, FTY720, or SEW2871. All the significances are compared to untreated PBMCs. Results summarize the results of at least four independent experiments. Results of Student’s *t*-test: *(P < 0.05, two-tailed test), ** (P < 0.01, two-tailed test), *** (P < 0.001, two-tailed test), **** (P < 0.0001, two-tailed test).

Among the S1P receptors, S1P1 is the most widespread receptor in the immune system and is expressed by several cell populations. S1P1 expression on the cell surface is downregulated in the presence of S1P [38]. Since AD2900 functions as an antagonist on S1P1, we next checked whether AD2900 affects S1P1 expression on PBMCs; FTY720 and SEW2817 were used as positive controls. The percentage of cells expressing S1P1 on their cell surface was detected by FACS analysis and was calculated as % of expression in non-treated cells. Low doses of AD2900 led to a decreased number of S1P1-expressing PBMCs after a 30-min treatment (Figure 3A and Supplementary Figure 2C). S1P1 expression was significantly and dose-dependently decreased by the 25–2000 nM of AD2900 treatment. The highest effect was obtained at 100 nM with a mean of 51% ± 9% expression as compared to untreated control PBMCs. At 4000 nM, S1P1 expression level returned to that of untreated control. The effect of AD2900 on S1P1 cell surface expression was transient and reversed shortly after a 60-min treatment (Figure 3B). SEW2871 had a similar effect, while FTY720 treatment led to smaller reduction of S1P1 cell surface expression, but its effect was not reversed after a 60-min treatment.

CCR7 is known as a lymph node homing receptor, and it is also involved in lymphocyte recirculation [39–41]. Together with S1P1, CCR7 controls the circulation of lymphocytes between the SLOs and the blood or lymph. We therefore examined the effect of AD2900 on the level of CCR7-surface-expressing PBMCs. Figure 3C and Supplementary Figure 2D demonstrate a small but significant reduction, 22% ± 5%, of the normal percentage of CCR7-expressing cell population upon a 30-min treatment with AD2900. Other analogues did not show any significant effects on CCR7 expression. Taken together, these data demonstrate a specific effect for the S1P analogue AD2900 on the populations of S1P1- and CCR7-expressing cells, which implies a possible influence on lymphocyte circulation.

### AD2900 administration influences white cell counts in healthy mice

S1P receptors have a major role in cell migration. For example, animal studies showed a crucial role for S1P1 in lymphocyte trafficking and specifically in lymph node egression [5, 17, 20, 30, 42]. AD2900 is an antagonist of S1P receptors, and it significantly downregulates the expression of S1P1 and CCR7 in PBMCs *in vitro*. Based on these findings, we aimed to investigate whether AD2900 plays a role in lymphocyte localization *in vivo*.

First, to confirm that AD2900 also inhibits murine lymphocyte proliferation, the MLR experiment was repeated using murine splenocytes. Mice T-cell proliferation was significantly and dose-dependently inhibited after a 1-day pretreatment with AD2900 following 3-day activation in mice MLR test. Proliferation was suppressed by AD2900, with a mean of up to 73% ± 3% inhibition at the highest concentration (Supplementary Figure 4A).

**Figure 4:**
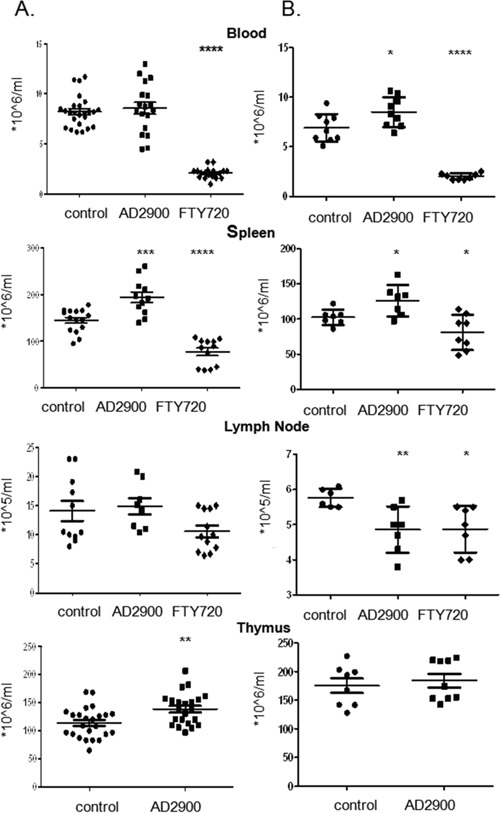
AD2900 treatment alters the distribution of leukocytes in the thymus, blood, spleen, and peripheral lymph nodes in mice C57BL/6 female mice were orally administered with 1.8 mg/l AD2900 in their drinking water for 2 days **(A)** or 7 days **(B)**, and FTY720 (1.8 mg/l) was used as a positive control. Leukocytes from the thymus, blood, spleen, and peripheral lymph nodes (pLNs) were collected and counted. All the significances are compared to untreated healthy mice. Results summarize four independent experiments in figure **(A)** and two independent experiments in figure **(B)**. n = 4–7 mice per group in each experiment. Results of Student’s *t*-test:*(P < 0.05, two-tailed test), ** (P < 0.01, two-tailed test), *** (P < 0.001, two-tailed test), **** (P < 0.0001, two-tailed test).

Next, 7- to 8-week-old C57BL/6J female mice were orally administered with AD2900 mixed in drinking water in different dosages (1.8, 2.7, or 3.6 mg/l) for 2 days (Figure 4A) or 7 days (Figure 4B); FTY720 was used as a positive control. In the 7-day experiment, the water was replaced on Day 3 with fresh water containing the same concentration of analog. At the end of the experiment, blood, spleen, inguinal lymph nodes, and thymus were obtained from each animal and the number of leukocytes was evaluated. At the dosage of 1.8 mg/l, the 7-day treatment with AD2900 significantly increased mice WBC count by 23% ± 7%. Both 2-day and 7-day treatments with AD2900 significantly increased the splenocyte counts by 33% ± 9% and 20% ± 7%, respectively. The 2-day treatment with AD2900 significantly increased the thymus WBC count by 25% ± 8.2%. On the other hand, AD2900 treatment significantly reduced the number of murine leukocytes by 18% ± 4% of that of the control in the lymph nodes after the 7-day treatment. As expected, FTY720 treatment dramatically reduced both the WBC and splenocyte counts after both 2-day and 7-day treatments but showed only minor effects on the lymph node leukocyte count.

### AD2900 administration affects the distribution of murine lymphocyte populations

**Figure 5:**
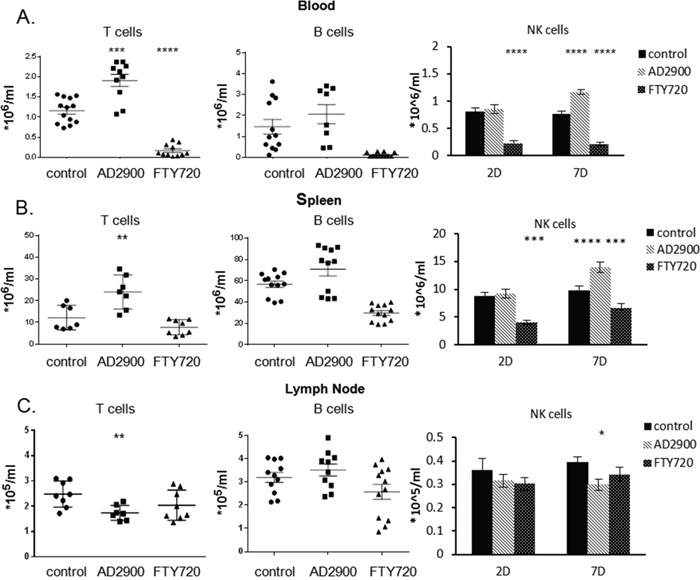
AD2900 treatment affects the distribution of T cells, B cells, and NK cells in blood, spleen, and peripheral lymph nodes C57BL/6 mice were orally administered with AD2900 or FTY720, as shown in Figure 4. Leukocytes from blood, spleen, and pLNs were collected and stained with TCR-β, CD19, and CD49b fluorescent antibodies and then analyzed by FACS analysis. The percentages of TCR-β+ T cells (left panel), CD19+ B cells (central panel), and CD49b+ NK cells (right panel) of the total leukocytes from blood **(A)**, spleen **(B)**, and pLNs **(C)** are shown. The results of T and B cells are from 2 days’ experiments. The results of NK cells are from 2 and 7 days’ experiments. All the significances are compared to untreated healthy mice. Results summarize three independent experiments. Results of Student’s *t*-test:*(P < 0.05, two-tailed test), ** (P < 0.01, two-tailed test), *** (P < 0.001, two-tailed test), **** (P < 0.0001, two-tailed test).

S1P receptors are differentially expressed by distinct immune cell populations. Since AD2900 is an antagonist to all S1P receptors, we next evaluated its influence on the localization of different murine lymphocyte populations. To achieve this aim, we performed FACS analysis of the blood (Figure 5A), spleen (Figure 5B), and lymph node cells (Figure 5C) using antibodies against surface markers of T cells, B cells, and NK cells (TCR-β, CD19, and CD49b, respectively). After a 2-day treatment with 1.8 mg/l AD2900, the average count of T cells in the blood was significantly increased by 81.9% (0.7488 ± 0.1641× 10^6^ cells/ml). The average NK cell count was significantly increased by 52.1% (0.3987 ± 0.0706 × 10^6^ cells/ml) only after a 7-day treatment with AD2900. In the spleen, the average count of T cells was also significantly increased by 96.7% (11.79 ± 3.64 × 10^6^ cells/ml) after a 2-day treatment. The NK cell count was elevated by 55.7% (4.741 ± 0.8621 × 10^6^ cells/ml) after a 7-day treatment with AD2900. On the other hand, in the lymph nodes, after a 2-day treatment, the T-cell average count was significantly decreased by 30% (0.7411 ± 0.2217 × 10^5^ cells/ml); the NK cell count was significantly decreased by 20.8% (0.07852 ± 0.03572 × 10^5^ cells/ml) after a 7-day treatment, and the B cell count was not significantly influenced in all the organs. These results indicate that leukocyte count alterations in the blood, spleen, and lymph nodes upon treatment are primarily attributed to the T- and NK cell populations. The trend in the B-cell change was opposite to that in the T- and NK cell changes in the lymph nodes.

### The influence of AD2900 on the localization of different T-cell subpopulations

Migration of T cells is crucial for an effective effector response to occur. The initiation of naïve T-cell activation usually occurs in the lymph nodes [13, 14]. In systemic infections, responses are also initiated in the spleen where antigen-presenting dendritic cells enter from the blood [43].

**Figure 6:**
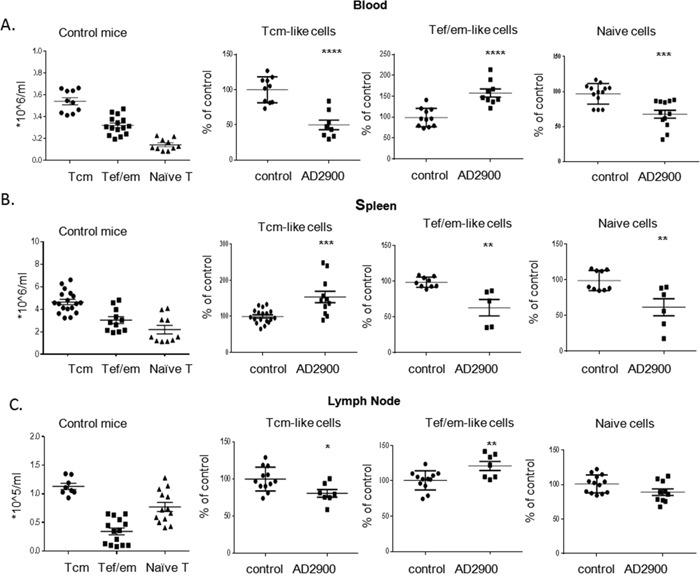
AD2900 treatment influences the distribution of mice T-cell populations in the blood, spleen, and peripheral lymph nodes C57BL/6 mice were orally administered with AD2900 or FTY720, as shown in Figure 4. Leukocytes from blood, spleen, and pLNs were collected and stained with TCR-β, CD44, and CCR7 fluorescent antibodies and then analyzed by FACS analysis. The percentages of CCR7+ CD44+ Tcm-like cells (left panel), CCR7-CD44+ Tef/em-like cells (central panel), and CCR7 + CD44-naive T cells (right panel) of the total T-cell population (TCR-β+) in blood **(A)**, spleen **(B)**, and pLNs **(C)** are shown. All the significances are compared to untreated healthy mice. Results summarize three independent experiments. Results of Student’s *t*-test:*(P < 0.05, two-tailed test), ** (P < 0.01, two-tailed test), *** (P < 0.001, two-tailed test), **** (P < 0.0001, two-tailed test).

We have shown that AD2900 has a unique effect on T-cell distribution in mice. Since T-cell subpopulations such as naïve T cells, central memory T cells (Tcm), and effector/effector memory T cells (Tef/em) have designated roles in the immune response, the influence of AD2900 on the localization of these subpopulations was investigated. The analysis of T-cell subpopulations was performed by FACS using antibodies against commonly accepted surface markers. Some of the molecules that we used as markers are involved in T-cell migration, and their expression can be affected by our treatment. For example, our *in vitro* experiments demonstrated that AD2900 can significantly downregulate CCR7 surface expression. Therefore, we refer to the CCR7-CD44+ T-cell population as Tef/em-like cells and to the CCR7+ CD44+ (Figure 6) or CD44+ CD62L+ (Supplementary Figure 4B) T-cell population as Tcm-like cells. The calculated cell number of the different subpopulations in control mice is shown in the left panel of Figure 6.

We found that the population of naïve T cells (CCR7+CD44-) is significantly reduced by AD2900, with a mean of up to 32% ± 6% of that in the control in the blood and 38% ± 12% of that in the control in the spleen (Figures 6A and 6B). In the lymph nodes, the percentage of naïve T-cell population among all T cells remained the same as in that in the untreated condition (Figure 6C). However, in view of the fact that the T-cell population was significantly reduced, the number of naive T cells was decreased. On the other hand, the percentage of Tef/em-like cells in the blood was significantly upregulated by 48% ± 1% (Figure 6A). Similar to that in the blood, the T-cell population in the spleen was significantly increased. Within this population, the percentage of Tcm-like cells was significantly upregulated compared to that in the control (Figure 6B and Supplementary Figure 3B). This subpopulation was significantly downregulated in the blood and lymph nodes compared to that in the control (Figures 6A, 6C, and Supplementary Figure 4B). Taken together, these results indicate that AD2900 treatment leads to a significant reduction in the number of naïve T cells at the periphery and accumulation of Tef/em-like cells in the blood and Tcm-like cells in the spleen.

### AD2900 administration affects S1P1- and CCR7-expressing T-cell populations *in vivo*

**Figure 7:**
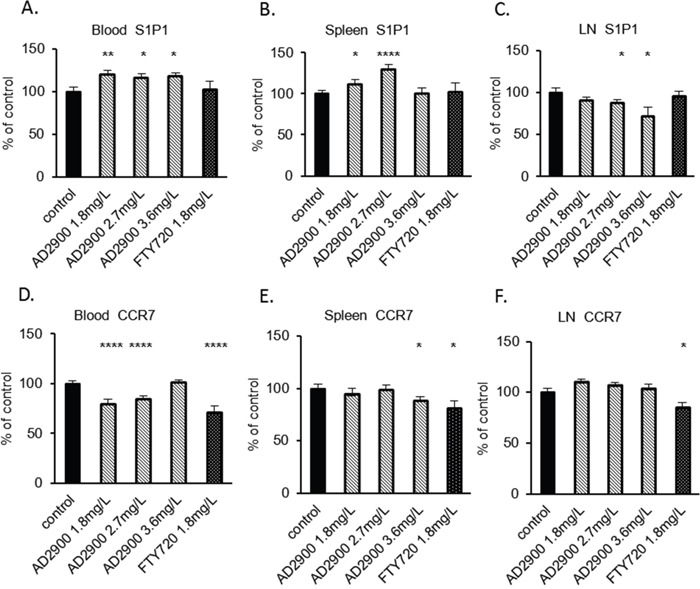
The influence of AD2900 on S1P1- and CCR7-positive T-cell populations in blood, spleen, and peripheral lymph nodes C57BL/6 mice were orally administered with 1.8, 2.7, and 3.6 mg/l AD2900 or 1.8 mg/l FTY720 for 2 days, as shown in Figure 4. Leukocytes from blood, spleen, and pLNs were collected and stained with CD3e and S1P1 or CCR7 fluorescent antibodies and then analyzed by FACS analysis. The percentages of S1P1+ CD3e+ T cells from blood **(A)**, spleen **(B)**, and pLNs **(C)** are shown. The percentages of CCR7+ CD3e+ T cells from blood **(D)**, spleen **(E)**, and pLNs **(F)** are shown. All the significances are compared to untreated healthy mice. Results summarize at least three independent experiments. Results of Student’s *t*-test:*(P < 0.05, two-tailed test), ** (P < 0.01, two-tailed test), *** (P < 0.001, two-tailed test), **** (P < 0.0001, two-tailed test).

The S1P gradient is a crucial factor for lymphocytes to egress from lymphoid tissues and re-enter the circulation. Lymphocyte S1P1 is downregulated in the blood, upregulated in lymphoid organs, and downregulated again in the lymph owing to the S1P gradient among different compartments [17]. Our *in vitro* results showed that AD2900 reduces the S1P1- and CCR7-positive populations. To identify whether the effect of AD2900 on T-cell localization may be mediated by an influence on S1P1 and CCR7 expression, we tested the percentage of S1P1- and CCR7-positive T-cell populations in the blood, spleen, and lymph node in each mouse after a 2-day treatment with AD2900. The percentage of cells expressing S1P1 or CCR7 on their cell surface was detected by FACS analysis and was calculated as % of expressing population in non-treated animals. The mean population of S1P1-positive T cells in the blood was significantly increased at dosages of 1.8, 2.7, and 3.6 mg/l by 20% ± 4%, 16% ± 5%, and 18% ± 4% of that of normal expression levels, respectively (Figure 7A). Regarding the spleen T cells, the S1P1 population was also significantly increased by 12% ± 5% and 31% ± 5% at dosages of 1.8 and 2.7 mg/l, respectively (Figure 7B). On the other hand, in the lymph nodes, the S1P1 population was significantly decreased by 12% ± 3% and 18% ± 11% at dosages of 2.7 and 3.6 mg/l, respectively (Figure 7C). FTY720 treatment did not demonstrate any effects on the S1P1 population in murine blood, spleen, and lymph nodes in our experiments (Figures 7A, 7B, and 7C). These results demonstrate that the effect of AD2900 on the expression of S1P1 on T cells is opposite to the S1P gradient. AD2900 elevates the S1P1-expressing population in the blood and spleen, whereas it reduces this population in the lymph nodes. The mean population of CCR7-positive T cells in the blood was significantly decreased at dosages of 1.8 and 2.7 mg/l by 21% ± 5% and 15% ± 4% of the normal expression levels, respectively (Figure 7D). Regarding the spleen T cells, the S1P1 population was significantly decreased by 12% ± 4% only at the highest dose of 3.6 mg/l (Figure 7E). In the lymph nodes, the S1P1 population was not affected by AD2900 treatment (Figure 7F). On the other hand, FTY720 treatment reduced the CCR7 population in murine blood, spleen, and lymph nodes by 15%–20% (Figures 7D, 7E, and 7F). These results are in correlation with the human PBMC results.

## DISCUSSION

Sphingosine-1-phosphate (S1P), a lipid mediator, is now considered to be a major regulator of immune cell trafficking and activation [1, 2] and is also involved in the progression of different malignancies [7, 8, 10, 44, 45]. Majority of the S1P actions are mediated by five members of the G protein-coupled S1P receptors (S1P1–S1P5). Among these receptors, S1P1 was shown to be involved in lymphocyte egression from the thymus and peripheral lymphoid tissues into the circulation [15, 18–20]. In this study, we report that one sphingolipid analogue, AD2900, inhibits lymphocyte activation and affects lymphocyte localization in a steady state. Proliferative response of activated PBMCs was dose-dependently inhibited by AD2900, while non-activated cells were not affected. Part of this effect may be attributed to apoptosis. However, the inhibitory effect of AD2900 is signal transduction dependent since it could be reversed by the addition of the intracellular [Ca^2+^]i chelator-BAPTA-AM or exogenous cAMP-8-bromo-cAMP to the culture during the activation. In T cells, S1P1, and S1P4 are the most important S1P receptor subtypes. S1P1 joins with the Gi/o protein, while S1P4 couples with both Gi/o and G12/13. Signaling through Gi/o can result in multiple functional effects. For example, it can lead to the activation of protein kinase C (PKC) and PLC to increase [Ca^2+^]i. Furthermore, signaling through Gi/o can inhibit adenylyl cyclase (AC) activity to reduce cAMP. Signaling through G12/13 can promote activation of the small GTPase Rho and the rho-associated kinase (ROCK) [46]. Our results suggest that Gi/o signaling is involved in the suppression of T-cell proliferation by AD2900. In transfected cells, S1P1 induction of [Ca^2+^]i mobilization followed PLC activation [47, 48]. S1P suppression of T-cell proliferation is also Ca^2+^ and PLC dependent, as shown by Glenn Dorsam et al [11] and in our results (Figure 2C). However, AD2900 suppression of T-cell proliferation involves [Ca^2+^]i mobilization but not PLC activation, indicating that PLC-independent pathways such as sphingosine kinase (SphK)/S1P contribute to this effect [49].

The viability of several human cancerous T-cell lines was also specifically affected by AD2900. Acute lymphoblastic leukemia is common in children and adults. The sensitivity of cancerous T cells, including T-ALL cells, to AD2900 suggests that this compound may be tested as a drug for treatment of patients with leukemia or lymphoma in the future.

We demonstrated that AD2900 is an antagonist to S1P receptors 1–5. Specifically, AD2900 shows high antagonistic activity to S1P5. Thus, we believed that AD2900 can have a broad influence on different cells within the immune system. AD2900 also affects the S1P1 surface expression on PBMCs. Studies have shown that the S1P gradient facilitates T-lymphocyte entry and egression between secondary lymphoid organs (SLOs) and the blood or lymph through the regulation of S1P1 expression on the surface of lymphocytes [15, 16, 20]. The egression of naive T cells from the thymus also depends on the signaling of S1P1 [20]. S1P receptors are involved in the migration of other immune cells. It was demonstrated that S1P/S1P5 facilitates NK cell egression from the bone marrow and lymph nodes [50, 51], while S1P/S1P1 induces marginal zone B-cell migration into follicles in the spleen [52]. Indeed, we observed a distinctive effect of AD2900 treatment on the localization of T, B, and NK cells *in vivo*. Interestingly, although AD2900 is a strong antagonist of S1P5, which is primarily expressed on NK cells, the influence of AD2900 on NK cell localization is seen only after a longer period of time as compared to the effect on T-cell localization. In the literature, however, the significance of S1P5 for NK circulation *in vivo* was tested using knockout mice and FTY720 treatment [50, 51], and therefore, the actual timing required for the effect of S1P gradient on NK cell migration is not known. Our results with FTY720 are consistent with previous studies. Altogether, these results suggest that FTY720 treatment does not deplete NK cells from blood circulation [51, 53]. Conversely, Jenne CN et al claimed that NK cell exit from LNs is sensitive to FTY720 [50].

AD2900 treatment had a dramatic effect on T-cell localization, which is opposite to the effect of FTY720; the cells accumulated in the blood and did not enter the lymph nodes. Since S1P1 is important for lymphocyte recirculation and the regulation of T-cell egression from the thymus and peripheral lymphoid organs [12, 15, 17, 18, 20, 21, 54], we measured the size of S1P1 receptor-positive population. AD2900 treatment caused a transient and reversible decrease in S1P1-positive human PBMC population *in vitro*. The CCR7 expression determines the ability of lymphocytes to enter lymph nodes, allowing naïve T cells to encounter the specific antigens presented by APC [39–41, 55, 56]. Our results show, both *in vitro* and *in vivo*, that the CCR7-positive population in blood cells is also decreased by AD2900 treatment. Therefore, we suggest that AD2900 can influence the ability of T cells to enter the lymph nodes through its effects on both S1P1 and CCR7. Indeed, our *in vivo* results revealed that AD2900 treatment partially reversed the effect of S1P gradient on S1P1-positive T-cell population; S1P1 population was increased in the blood and spleen but decreased in the lymph nodes. It is possible that as an antagonist, AD2900 competes with S1P to bind S1P1. In turn, it can block S1P1 downstream signaling and generate a conditional S1P1 knockout in the mice. Unlike AD2900, the effect of FTY720 on S1P1 expression *in vitro* was not reversed after 1-hour treatment. This could mean that in contrast to FTY720, which induces S1P1 receptor degradation, and more like SEW2817 and S1P [24, 38, 57], AD2900 may induce S1P1 internalization and recycling. The effect of AD2900 on S1P1 surface expression is different from the effect of other described S1P antagonists, which do not reduce surface S1P1 [58, 59]. However, although most of the antagonists cause long-term lymphopenia [58, 60, 61], the long-term effect of the specific S1P1 antagonist W146 on lymphocyte counts is similar to the effect of AD2900. These combined data lead to the conclusion that different antagonists interfere with S1P signaling at different stages, and therefore, their effect on the immune cells varies.

T cells can be divided to distinct subpopulations, which fulfill different functional needs. In our study, we found that the numbers of naïve T cells in the blood, lymph nodes, and spleen were significantly decreased upon treatment with AD2900. Matloubian M et al have shown that both CD4+T cells and CD8+T cells accumulated in the thymus in S1P1^−/−^ knockout mice, leading to a decreased number of T cells in the circulation [20]. As an antagonist, AD2900 may cause the attenuated egression of naïve T cells from the thymus to the circulation and the accumulation of these cells in the thymus, which in turn decreases the naïve T-cell counts in the circulation and peripheral lymphoid tissues. Indeed, our results showed increased thymus WBC count in the AD2900-treated mice. In addition, it is possible that some naïve T cells lost their CCR7 expression due to the treatment and were therefore not counted as CCR7+CD44-naïve cells in our analysis. Such cells will not be able to enter the lymph nodes.

The localizations of two other T-cell subpopulations were examined, Tef/em-like cells and Tcm-like cells. In the blood of AD2900-treated mice, the percentages of both Tcm-like cells and naïve T cells were significantly decreased. Consequently, the Tef/em-like cells were the primarily upregulated population. The Tef/em-like cells are characterized as TCR-β+CCR7-CD44+ cells [62–64]. Because these cells lack CCR7, they do not respond to CCL19 signals from the lymph nodes or spleen. Together with the upregulation of S1P1 on the blood T cells, this explains the accumulation of Tef/em-like cells in the blood.

In the spleen of AD2900-treated mice, we found that the percentages of both Tef/em-like cells and naïve T cells were significantly decreased, and the percentage of Tcm-like cells was significantly increased. Tcm-like cells (marked as TCR-β+CD62L+CD44+) express CCR7 [64], and are therefore attracted to CCL19, secreted in the T-cell zone of the spleen [56, 65]. While most of the studies on S1P signaling in the spleen concentrated on B cells [52, 66], it was also shown that the entry of T cells from the marginal zone to the T-cell zone in the spleen is S1P dependent [52]. Because AD2900 competes with a relatively high concentration of S1P in the marginal zone of the spleen, leading to the upregulation of S1P1, we suggest that Tcm-like cells accumulate in the marginal zone without entering the T-cell zone.

**Figure 8:**
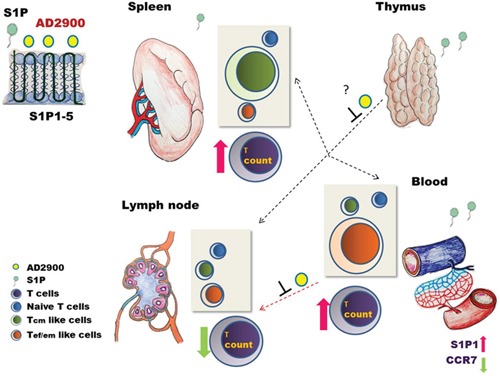
A model for the intervention of AD2900 in the localization of murine T lymphocytes As an antagonist to S1P receptors 1–5, AD2900 can compete with S1P to bind S1P receptors leading to reduced S1P signaling and enhanced expression of S1P1 on T cells in S1P-rich environments such as the blood and the spleen. This altered expression, together with decreased CCR7 expression, inhibits T-cell entry into the lymph nodes (LNs) from the blood, causing accumulation of T cells in the blood. However, the entry of T cells to the spleen is not affected because it is not S1P dependent. Since Tcm-like cells express CCR7, these cells are attracted to the spleen and accumulate in it; yet, S1P1 elevated expression may have an effect on the S1P-dependent ingression of these cells from the MZ to the white pulp. Tef/em-like cells, which are CCR7 negative, are the primary T-cell subpopulation in the blood after AD2900 treatment. The significant decrease in naive T-cell counts in the circulation and peripheral lymphoid tissues tested may be explained by the inhibition of S1P signaling in the thymus leading to attenuated T-cell egression from the thymus to the circulation. Arrow key: thick = response; dashed = inhibition.

Considering both the *in vitro* and *in vivo* results, we propose a model for AD2900-mediated effects on T-cell localization (Figure 8). As an S1P receptor antagonist, AD2900 can compete with S1P, which exists at a high concentration in the blood and the marginal zone of the spleen, to bind S1P receptors and reduce S1P signaling. This is accompanied by the upregulation of S1P1 on both blood and spleen T cells. The entry of T cells into the lymph nodes, which is dependent on the S1P gradient, is impaired and T cells accumulate in the blood and spleen. The naïve T cells, however, are also decreased in the blood and spleen of AD2900-treated mice, possibly because of the effect of the drug on the thymus egression. The distribution of Tef/em-like cells and Tcm-like cells between the spleen and blood is based on the differential expression of CCR7 in these cells. T cells can enter the spleen freely from the blood, but the entry of T cells to the T-cell zone is dependent on S1P and CCL19/21. Consequently, the CCR7-positive Tcm-like cells are attracted to the spleen and accumulate in this organ, while the CCR7-negative Tef/em-like cells accumulate in the blood.

Taken together, these data reveal that AD2900, an S1P1-5 antagonist, is a potent S1P analogue. Its direct anti-inflammatory effects coupled with its effects on lymphocyte localization, especially the inhibition of T-cell entry into the lymph nodes, make AD2900 as a potential immunomodulatory and anti-inflammatory drug. The inhibitory effect of AD2900 on the viability of cancerous T-cell lines suggests a possible use of AD2900 for the treatment of hematological malignancies. The involvement of S1P1 signaling in the migration of leukemia and lymphoma cells has been demonstrated [8, 67], and the effect of AD2900 on malignant cells might be similar to its effect on lymphocytes. The clinical effect of AD2900 should be further investigated in animal models of inflammatory diseases.

## MATERIALS AND METHODS

### Synthesized sphingolipid analogues and commercial analogues

AD2900, AD2730, AD2750, YZ297, and YZ158 were synthesized in our laboratory [36]. Fingolimod, hydrochloride salt (FTY720), was purchased from LC laboratories (MA, USA). SEW2871 was purchased from Caymen Chemical (MI, USA). All the compounds were dissolved in DMSO and stored at −80°C. Sphingosine-1-phosphate (S1P) was purchased from Sigma-Aldrich (Rehovot, Israel) and was prepared as a 1 mg/ml stock solution in methanol. Et-18-OCH3, BAPTA-AM, and 8-bromo-cAMP were obtained from ENZO life sciences (NY, USA) and dissolved according to the manufacturer’s instructions. Prior to the experiment, the compounds were diluted in culture medium.

### Sphingosine-1-phosphate receptors 1–5 (S1P1–5) agonists and antagonist test

The S1P1 agonist test and S1P2, S1P3, S1P4, and S1P5 agonist and antagonist tests were conducted as previously described using S1P1 CRE-bla CHO cells, S1P2 CRE-bla CHO cells, S1P3 NFAT-bla CHO cells, Tango™ S1P4 (EDG6)-bla U-2 OS cells, and Tango™ S1P5 (EDG5)-bla U-2 OS cells [37]. The S1P1 antagonist assay was carried out according to the manufacturer’s instructions using a Tango™ S1P1 (EDG1)-bla U-2 OS Cell-Based Assay (Invitrogen, NY, USA). Assay cutoff: Compounds with an EC50 of ≤10 mM were considered “active.” Compounds with an EC50 of >10 μM were considered “inactive.” CHO: Chinese Hamster Ovary cell line, bla: beta-lactamase, CRE: the cAMP response element, NFAT: nuclear factor of activated T-cell, EDG: endothelial barrier integrity, U-2 OS: human osteosarcoma cell line.

### T-cell proliferation assays

Human PBLs were collected from human whole blood collected from healthy donors. Subsequently, the cells were centrifuged on a Ficoll-Paque gradient (Fresenius Kabi Norge AS, Oslo, Norway) and were isolated from the interphase layer. For the human MLR experiment, PBMCs from one healthy donor were pretreated for 1 day with AD2900, AD2730, AD2750, YZ297, YZ158, FTY720, or SEW2871. After 24 h, cells were washed and plated at a concentration of 5 × 10^4^ cells/well in round bottom 96-well plates containing RPMI-1640 medium Supplemented with 10% human AB serum, 1% penicillin/streptomycin, and 1% l-glutamine (Beit Haemek, Israel). PBMCs from a second donor were irradiated at 3000 γ dosage and added to the plate in a 1:1 ratio with the first donor cells. The cells were cultured for 5 days. For the thymidine incorporation assay, after 4 days of co-culture, cells were pulsed with ^3^H-thymidine at 1 μCi (0.037MBq) per well (PerkinElmer, MA, USA) for 16 additional hours and then harvested onto glass-fiber filters. ^3^H-thymidine incorporation was measured using Top Count. NXT (PerkinElmer, UK). In other experiments, the supernatant was harvested after 5 days of co-culture for IFN-γ and TNF-α cytokine ELISA measurements (eBioscience, CA, USA). The cells were then collected for assessing apoptosis by FACS in MACSQuant analyzer (Miltenyi Biotech, Germany). In another set of experiments, the PBMCs of two donors were mixed. Sphingolipid analogues were then added to the culture and sustained through the 5 days of the MLR experiment.

### Cancerous cell viability assay

Jurkat, CEM/C1, Molt4, and H9 cells were purchased and grown according to the ATCC recommendations for the study using RPMI-1640 medium Supplemented with 10% FCS, 1% penicillin/streptomycin, and 1% l-glutamine (Beit Haemek, Israel). The cell viability test was performed using XTT-(2, 3-bis-(2-methoxy-4-nitro-5-sulfophenyl)-2H-tetrazolium-5-carboxanilide test (Sigma-Aldrich, Rehovot, Israel) according to the manufacturer’s protocol.

### FACS

Human PBMCs: For the apoptosis test, anti-AnnexinV-FITC (BD Biosciences, CA, USA) was used. To analyze S1P1 surface expression on AD2900-treated PBMCs, the following were used: anti-S1P1 (Alomone labs, Israel), anti-EDG-1 (Abcam, UK), Dylight405-conjugated AffiniPure Goat Anti-Rabbit lgG (H + L), and APC-conjugated AffiniPure F(ab')2 Goat Anti-Rabbit lgG(H + L) (Jackson ImmonoResearch, USA). For CCR7 expression, we used anti-CCR7-APC (Biolegend, CA, USA).

Murine lymphocytes: The phenotype of murine lymphocytes was analyzed by flow cytometry using anti-CD3e-FITC, anti-CD19-PB, anti-CCR7-APC, anti-CD44-FITC, anti-CD62L-APC, and anti-TCR-β-PB (Biolegend, CA, USA).

To eliminate the red blood cells, RBC lysis buffer (Beit Haemek, Israel) or BD FACS lysing solution (BD Biosciences, CA, USA) was added into murine blood and splenocyte samples. Flow cytometry was performed using the MACSQuant® Analyzer (Miltenyi Biotech, Germany), and the data were analyzed using FCS Express V3 software.

### *In vivo* experiments

C57BL/6 female mice aged 7–8 weeks were obtained from Harlan Laboratories (Jerusalem, Israel). The animal study was conducted under appropriate conditions and was approved by the Institutional Animal Care and Use Committee of the Hebrew University of Jerusalem in accordance with the national laws and regulations for the protection of animals. AD2900 was dissolved in 10% BSA (MP Biomedicals, CA, USA) in DDW at 1 mg/ml and then further diluted in 200 ml water to get final concentrations at 1.8, 2.7, or 3.6 mg/l. AD2900 and FTY720 were supplied ad libitum to the mice for 2 days or 7 days. At the end of the experiment, blood was collected, and the mice were sacrificed and tissues (lymph nodes and spleen) were harvested.

Murine blood samples, collected in tubes coated with EDTA (Grelner bio-one, Germany), were counted by a Mindray veterinary hematology Analyzer (Mindray, China). Single cell suspensions of lymph nodes and spleen were made in 1.5 ml of PBS. Cell counts were determined from these suspensions, yielding results in thousands of cells per microliter (μl).

### Statistical analysis

*In vitro* results represent mean values of at least three experiments. In each experiment, duplicate or triplicate samples were performed. p values were calculated using Student’s *t*-test. SDs and p values in all *in vivo* experiments were calculated using Student’s *t*-test using Prism software. P value < 0.05 was considered statistically significant.

## SUPPLEMENTARY MATERIALS FIGURES



## Abbreviations

S1P: Sphingosine-1-phosphate
PBMCs: Peripheral blood mononuclear cells
PLC: Phospholipase C
NK: Natural killer
T-ALL: T-cell acute lymphoblastic leukemia
Tcm: Central memory T cells
Tef/em: Effector/effector memory T cells
LD50: Lethal dose 50%
NMR: Nuclear magnetic resonance spectroscopy
AC: Adenylyl cyclase
ROCK: Rho-associated kinase
SphK: Sphingosine kinase
MLR: Mixed lymphocyte reaction
SLOs: Secondary lymphoid organs.
