# Supplementary material for: The novel sphingosine-1-phosphate receptors antagonist AD2900 affects lymphocyte activation and inhibits T-cell entry into the lymph nodes
Source: Oncotarget. 2017 Jun 27;8(32):53563–80. doi: 10.18632/oncotarget.18626 (PMC5581131; doi:10.18632/oncotarget.18626)
Supplement: Supplementary file 1 [file oncotarget-08-53563-s001.pdf]

The novel sphingosine-1-phosphate receptors antagonist AD2900 affects lymphocyte activation and inhibits T-cell entry into the lymph nodes

SUPPLEMENTARY MATERIALS

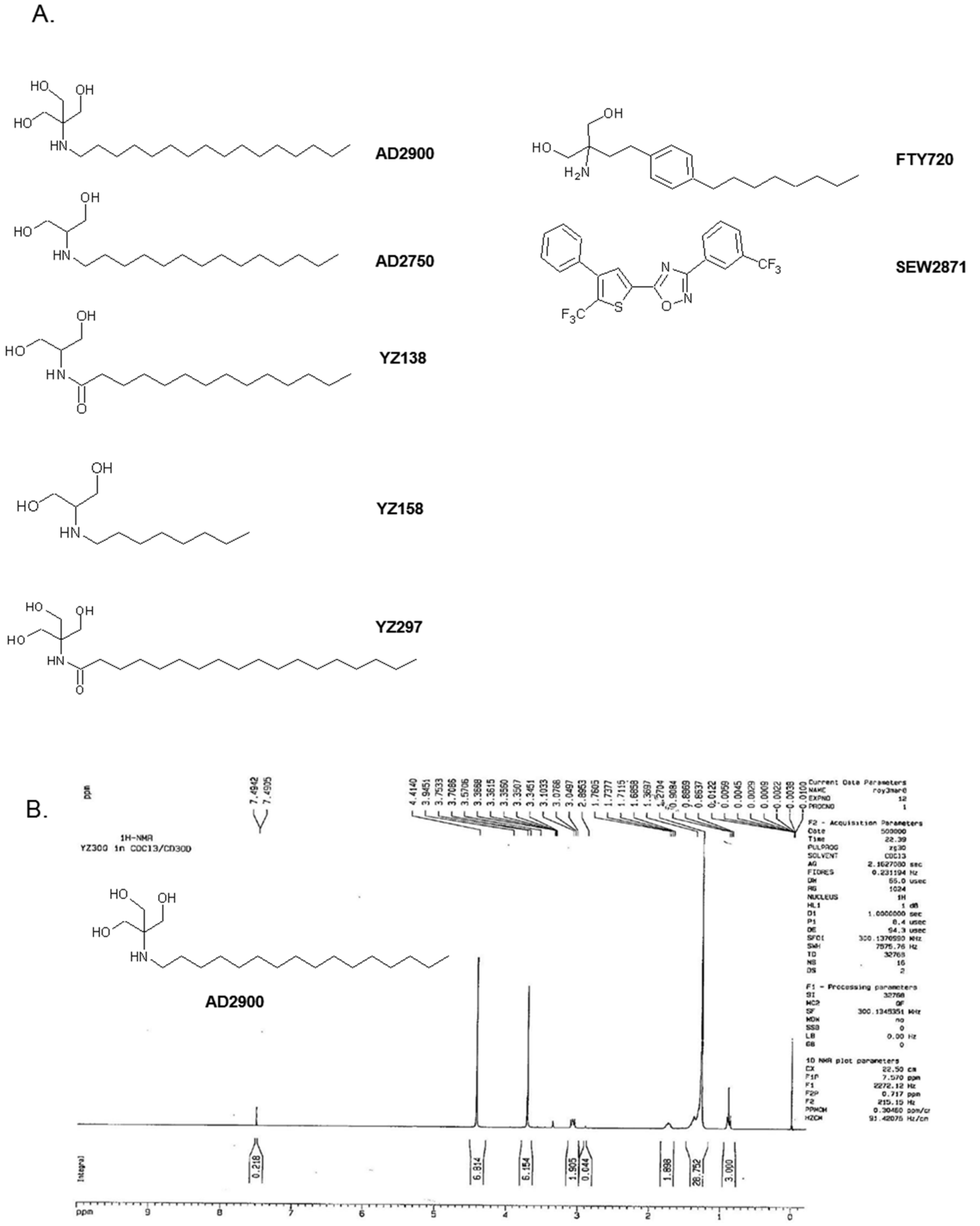

Supplementary Figure 1: (A) Chemical structures of AD2900, AD2750, YZ138, YZ158, YZ297, FTY720 and SEW2871. **(B)** Nuclear magnetic resonance (NMR) spectrum of AD2900.

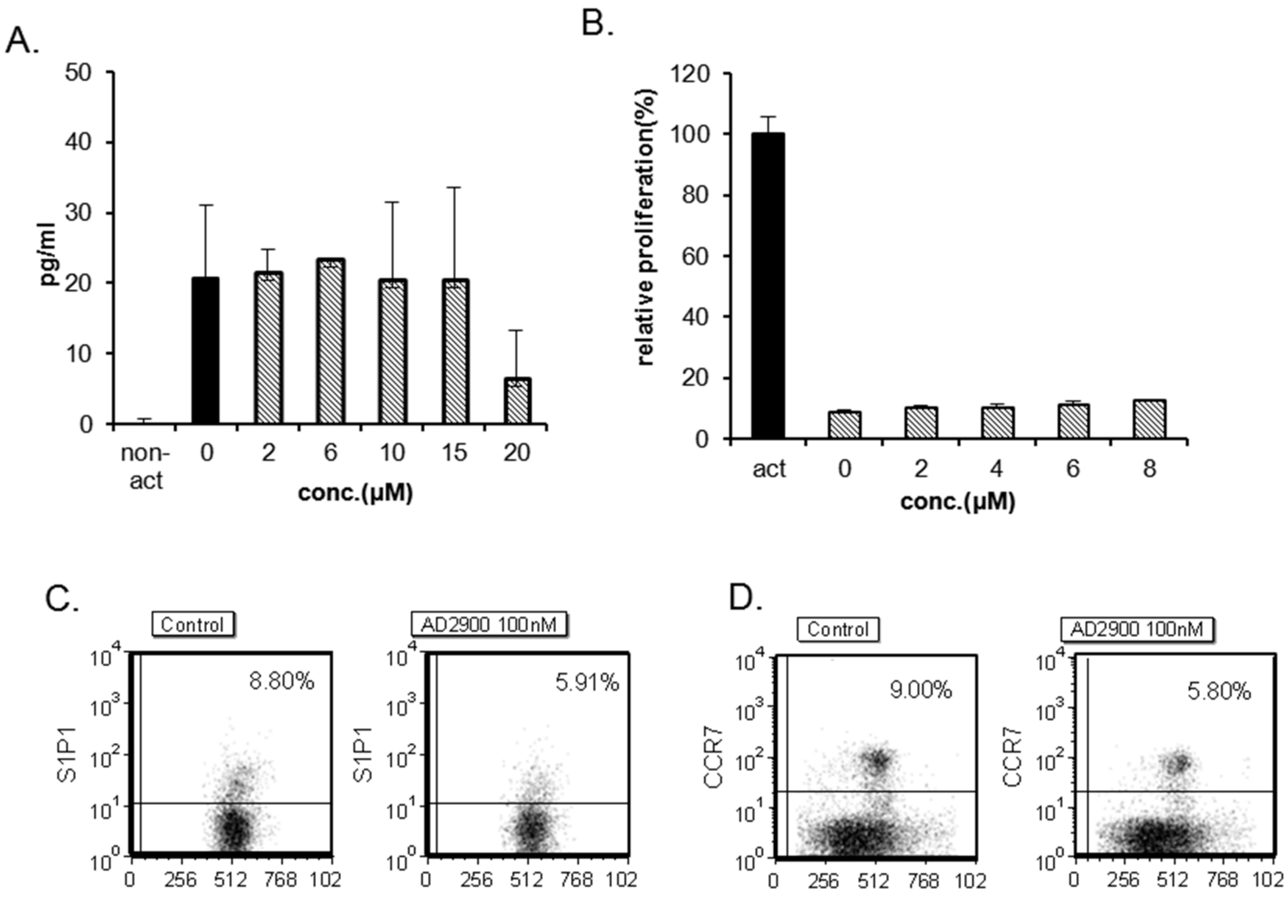

Supplementary Figure 2: AD2900 inhibition of activation is not associated with a change in TNFα secretion and doesn't inhibit non-activated human lymphocyte proliferation. **(A)** Cytokine secretion from activated PMMC was tested by ELISA analysis following one day AD2900 pretreatment in hMLR. At the 5th day of hMLR, supernatants were collected and assayed for the levels of TNF-α. **(B)** The levels of proliferative response of non-activated hPBL with different concentrations of AD2900 were tested. [3H] thymidine was incorporated for at least 16 hours at the 3rd day of the experiment. Significance is compared to untreated hPBL. Results summarize three independent experiments. **(C)** FACS analysis. S1P1 expression was tested in PBMCs after a 30-min treatment with 100 nM AD2900. **(D)** The percentage of CCR7-positive PBMCs was tested by FACS analysis after a 30-min treatment with 100 nM AD2900.

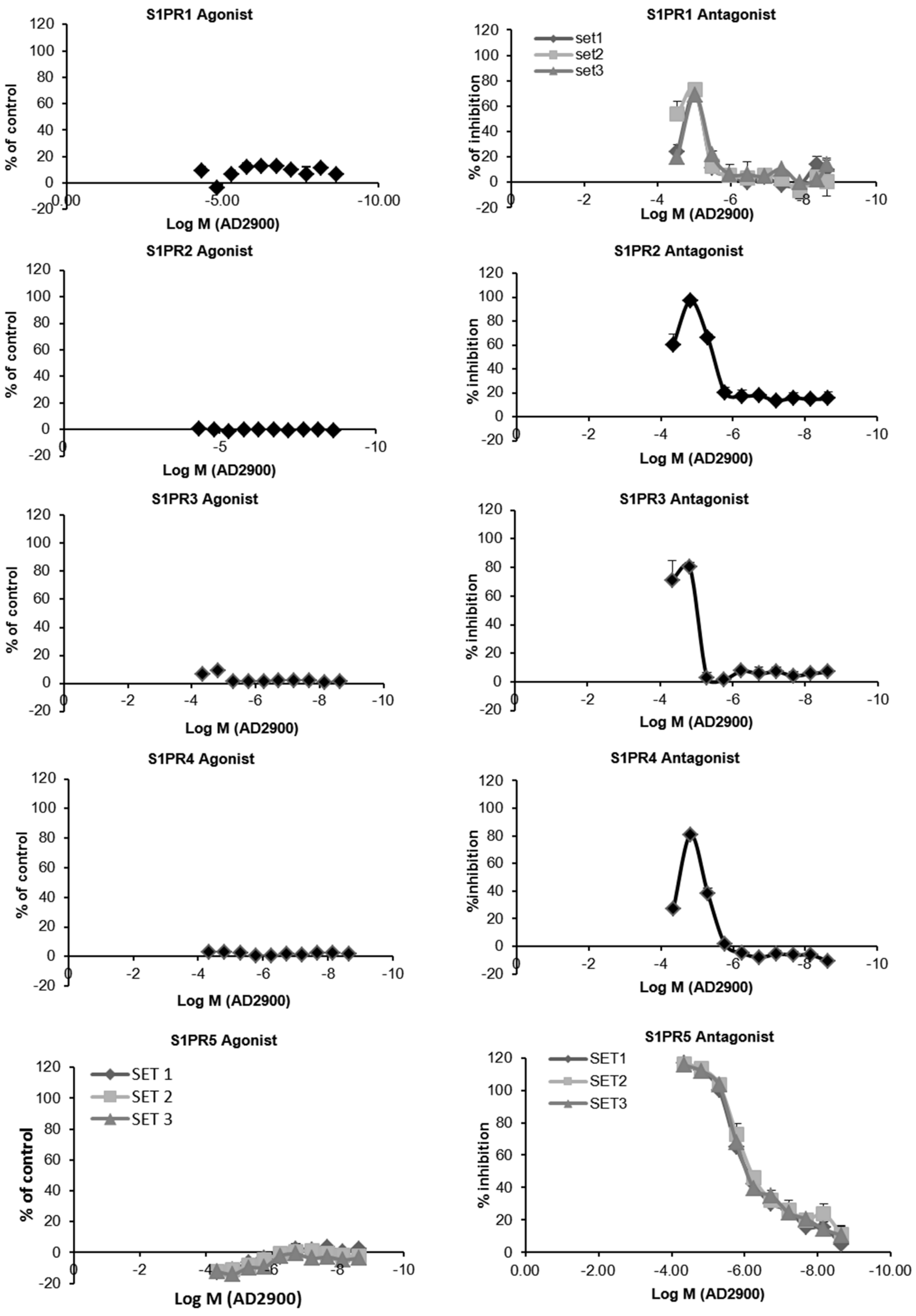

Supplementary Figure 3: AD2900 shows antagonistic activities against S1P1, 2, 3, 4 and 5. Antagonistic and agonistic properties of AD2900 on S1P1-5 were tested by using S1P1, S1P2 CRE-BLA CHO cell lines, S1P3 NFAT-BLA CHO cell line or S1P1, S1P4, S1P5 TANGO U-2 OS cell lines in a high-throughput screening experiment. IC50 values (µM) or EC50 values (µM) of AD2900 were measured. Data represented as mean 6 SEM obtained from three independent experiments. Abbreviations: CHO, Chinese Hamster Ovary; NFAT, nuclear factor of activated T-cell; BLA, beta lactamase; CRE, the cAMP Response Element promoter; U-2 OS, human osteosarcoma.

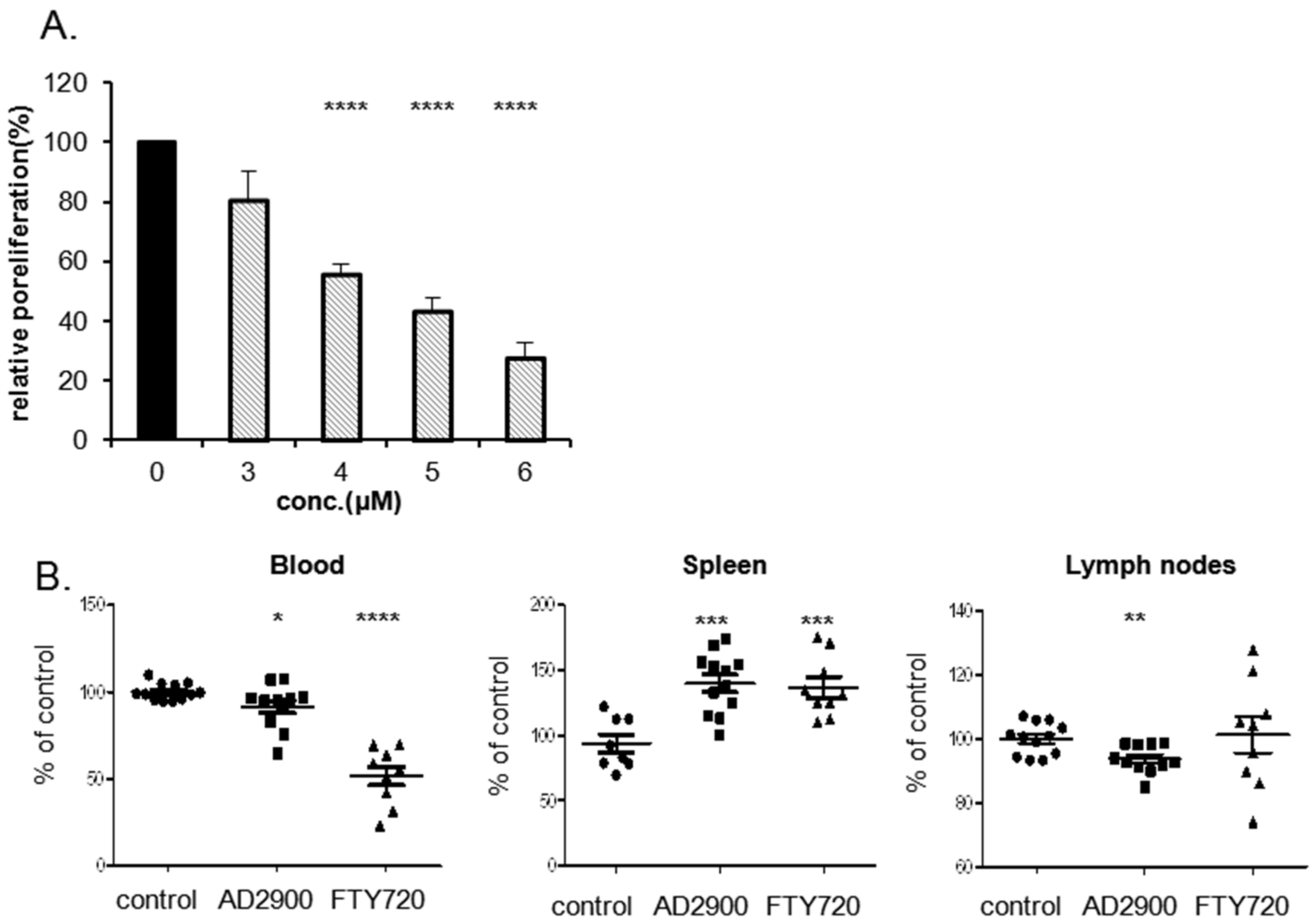

Supplementary Figure 4: AD2900 inhibits mice splenocytes proliferation in allogeneic activation *in vitro*. *In vivo*- the percentage of Tcm-like cells is up-regulated in the spleen of AD2900 treated mice. **(A)** The levels of proliferative response of mouse splenocytes after one day pretreatment with different concentrations of AD2900 were tested by mouse allogeneic mixed lymphocyte reaction (MLR) assay followed by a [3H] thymidine incorporation analysis. [3H] thymidine was incorporated for at least 16 hours at the 3rd day of the experiment. Significance is compared to untreated mice splenocytes. **(B)** C57BL/6 mice were orally administered AD2900 or FTY720. Leucocytes from blood, spleen and pLNs were collected and stained with TCR-β, CD62L and CD44 florescent antibodies and then analyzed by FACS analysis. The percent of CD62L+CD44+ (Tcm) like cells out of the total T cell population (TCR-β+) in blood, spleen and pLNs are shown. All the significances are compared to untreated healthy mice. Graphs summarize results of at least three independent experiments. Results of Student's t test :*($P < 0.05$, two-tailed test), ** ($P < 0.01$, two-tailed test), *** ($P < 0.001$, two-tailed test), **** ($P < 0.0001$, two-tailed test).
